# "The Hospital" Medical Book Supplement.—No. VI

**Published:** 1908-04-18

**Authors:** 


### "THE HOSPITAL" MEDICAL BOOK SUPPLEMENT.—No. VI.

CONTENTS.

**Medicine—**
A System of Medicine ... ... ... ... ... ... ... 71
Diseases of Occupation ... ... ... ... ... ... ... 71
**Surgery—**
The Operations of Surgery ... ... ... ... ... ... 72
**Gynæcology and Obstetrics—**
A Short Practice of Gynæcology ... ... ... ... ... 72
A Text-book of Treatment ... ... ... ... ... ... 73
**Ophthalmology—**
Ophthalmia Neonatorum, with Especial Reference to its Causation and Prevention ... ... ... ... ... ... 73
Tests and Studies of the Ocular Muscles ... ... ... ... 73
**Pharmacology and Therapeutics—**
The Chemical Basis of Pharmacology: an Introduction to Pharmacodynamics Based on the Study of the Carbon Compounds 74
**Pathology—**
The Essentials of Cytology ... ... ... ... ... ... ... 74
Medical Laboratory Methods ... ... ... ... ... ... 74

### MEDICINE.

A System of Medicine. By Eminent Authorities in Great Britain, the United States, and the Continent; edited by William Osler, M.D., F.R.S., Regius Professor of Medicine in Oxford University, etc., assisted by Thomas McCrae, M.D., F.R.C.P., Associate Professor of Medicine and Clinical Therapeutics in the Johns Hopkins University, Baltimore. Volume III. Pp. 960. (London: Henry Frowde and Hodder and Stoughton. Price 30s. net.)

The first 548 pages of this volume are devoted to the continuation of the "Infectious Diseases"; 372 pages cover the "Diseases of the Respiratory Tract," and 40 pages are occupied by a very full and detailed index to the volume. In a sense, however, a considerably greater part of the book than the above is concerned with matters pulmonary, for tuberculosis is dealt with under the heading "Infectious Diseases."

It is much easier to criticise than it is to create, and although there are many points upon which the volume is far from perfect, as a whole it is very readable and good. There are certain disadvantages shared by all large systems of medicine. Medical science is advancing so quickly that that which is the full extent of knowledge to-day is almost out-of-date to-morrow. Consequently new editions of such systems as that before us become necessary, and the practitioner can scarcely afford to buy a series of volumes which must be replaced in a comparatively short time if the primary object of the purchase—namely, that of being kept up-to-date—is to be secured. To take a concrete example: opsonins and vaccines are an entirely new development since the publication of the first edition of Sir Clifford Allbutt's System of Medicine; no system which does not include these subjects can now be regarded as up-to-date. It is necessary to buy another edition therefore. In short, on account of the great cost and the short life of a system of medicine written upon the present lines, the work is one to be possessed by libraries rather than by private individuals. Good though the volume before us is in many respects, and bulky though it is, there are many ways in which it lacks completeness. It is manifestly impossible to enter upon all of these here, but we will mention a few. Tuberculosis, for example, is dealt with in several chapters, and yet there is no account of the value of skiagraphy in the detection of phthisis in otherwise doubtful cases. The whole article upon tuberculosis, indeed, lacks proper proportions; its title would lead one to expect that all tuberculous lesions would be dealt with, but the great bulk of the chapters deal with phthisis only; skin tuberculosis, for example, is dismissed in a page and a-half. The chapter upon the treatment of tuberculosis is good, containing full details of everything from general hygienic measures to tuberculin. The treatment of some other conditions is incompletely described. It might be thought, for example, that a volume such as this would be the very place to find out all about Sclavo's serum in the cure of anthrax—but one is disappointed to find that no details are given as to whether a single dose of the serum suffices, or whether it should be repeated, and if so when, and in what dose, and how often! This applies to many of the other paragraphs upon treatment throughout the volume. Again, every practitioner knows how difficult of interpretation the physical signs of broncho-pneumonia may be, yet they are dismissed in half a page. Apart from such deficiencies in details, this volume is an important contribution to medical literature.

---

Diseases of Occupation. By Thomas Oliver, M.D., F.R.C.P., Physician to the Victoria Infirmary, Newcastle-upon-Tyne, and Professor of Physiology, Durham University. 8vo. Pages xix. + 427. Five figs. (London: Methuen and Co. Price 10s. 6d. net.)

This volume is one of "The New Library of Medicine" series, and it deals with most important subjects which have attained an increased degree of medico-legal significance since the passage of recent Acts of Parliament. The author has studied the questions upon which he writes not only in England, but also in France and in the Netherlands, and he brings before the profession much that is otherwise lost to it in the annual reports of the Chief Inspector of Factories. The subjects dealt with include the factors contributing to industrial diseases and accidents of every kind; occupation neuroses; the effects of over-fatigue; the effects of various trades, and the diseases of soldiers, sailors, and fishermen. There is a short chapter upon rescue work in mines; and the volume ends with a very fair index. The printing is large and clear, the paper thick but light, the binding good, and altogether we think that the author has produced a volume which, while of interest and use to all medical men, must be almost indispensable to medical officers of health and to those whose patients work to any extent in factories, workshops, or mines. Members of Parliament and all lay persons interested in schemes for the social and hygienic betterment of working men and women will also find the volume very useful.

### SURGERY.

The Operations of Surgery. By W. H. A. Jacobson, M.Ch., F.R.C.S., and R. P. Rowlands, M.S., F.R.C.S. (London: J. and A. Churchill. 2 vols. 42s. net.)

This, the fifth, edition of what is popularly known as "Jacobson's Surgery" has been greatly enlarged and in many ways amended. Mr. Jacobson has lost the services of his able collaborator, Mr. Steward, who was responsible for a part of the second volume of the last edition, and his place has been taken by one of the junior assistant surgeons of Guy's Hospital. It is difficult, however, to separate clearly the work of the two collaborators. Mr. Rowlands, we are told, "has made himself entirely responsible" for the sections dealing with the general surgery of the abdomen (Vol. II.), but so much of the senior's original work is retained in these sections that they can scarcely be looked upon as sufficiently indicative of the ability of the new author. In addition, Mr. G. Bellingham Smith, assistant physician to the gynæcological department at Guy's Hospital, has thoroughly revised, and indeed almost rewritten, the section on gynæcological operations, for which Dr. Dakin was responsible in former editions.

It is somewhat difficult to judge a work which is intended for senior students and "for those recently elected to hospital appointments." As a text-book the fifth edition in point of size is far inferior to its predecessors. Crispness and conciseness have been sacrificed to the addition of details which do not add materially to the value of the work. There is a great deal of unnecessary and often irritating repetition, and far too much space has been wasted. The result of this exuberance is two exceedingly bulky volumes, inconveniently big, weighty to hold and weighty to read. They are indeed nicely printed, and the text has been enriched with really helpful and valuable illustrations, many new wood blocks having been introduced, but they are far too bulky to be convenient text-books.

Coming to the separate sections, it is at once obvious that much new matter has been introduced, while at the same time the excellencies of former editions have been retained. In the first volume more space has been devoted to operations on the upper extremity. The interscapulo-thoracic amputation is fully described in an ably condensed chapter. The chief additions are, however, to be found in the sections dealing with the surgery of the brain, which are exhaustive and contain an excellent consideration of the indications for surgical interference in cases of intra-cranial tumours. All the other sections contain valuable additional matter, though here and there one notices omissions. Thus the authors do not mention bimanual expression of the tube in cases of intubation; Simon Kuster's operation is not described, though Estlander's, Schede's, and Delorme's are mentioned, and resection of the lung itself is not noticed; while no descriptions of Ballenger's and Killian's operations for deflection of the nasal septum are given. The authors have made use of the experience gained during recent wars in the section dealing with gunshot injuries of the abdomen, and the sections on appendicitis, gastric ulcer, intestinal operations, and operations on the stomach have been brought up to date. A much-needed though somewhat diffuse and incomplete chapter on operative interference in cases of chronic constipation is included in this volume. A good description is given of Mata's operation of aneurysmorrhaphy, and the operations on the lower limb have been brought up to date by fuller accounts of the methods used in treating deformities. The section on spinal anæsthesia, however, is by no means full, and should receive attention in the next edition. The surgery of the urino-genital organs has been rewritten, but the chapter on the pancreas has been very little modified, in spite of the large amount of new work. Similarly, too, those dealing with the spleen and liver are not fully up to date. Minor points are the frequent misspelling of foreign names (a glaring one is "Kocker"), and such grammatical absurdities as "neither . . . nor . . . have," "whether or no," and the frequent use of split infinitives. On the whole, very little use has been made of the work of foreign writers, and as the book stands it is mainly an exposition of English and American methods, thus lacking the catholicity which is so characteristic of text-books such as those of Réclus, Tillmanns, and von Bergmann.

We have dwelt at some length on these points, not in any carping spirit, but because they are errors which should be amended in future editions. Nothing, however, can detract from the excellence of the solid foundation which Mr. Jacobson laid so well many years ago. The edifice has been more lavishly ornamented and considerably enlarged, but after all its outstanding features are those that attracted readers in the older editions—the painstaking, impartial discussion of *pros* and *cons*, the detailed accounts of illustrative cases, and the trenchant remarks, aphorisms such as only a master with a large clinical experience could have formulated. For these features this work will always be prized, and its perusal will always be instructive. The pity is that in the present edition they have been somewhat obscured by redundancy, and—in the second volume especially—by a style of writing which is painfully reminiscent of the "scissors and paste" method.

### GYNÆCOLOGY AND OBSTETRICS.

A Short Practice of Gynæcology. By Henry Jellett, M.D., F.R.C.P.I., Gynæcologist and Obstetrical Physician to Dr. Steevens' Hospital. (London: J. and A. Churchill. Third edition. Price 12s. 6d. net.)

In this third edition of Dr. Jellett's "Practice of Gynæcology" the chapters dealing with operations have, we are told, been largely re-written. They are certainly of a high degree of excellence, for they are clear and terse, exhaustive, and remarkably well illustrated. The merit of these chapters, which fill 240 out of the 508 pages of the text, is, unfortunately, far above that of the other sections of the book; anyone to whom the latter are likely to be of use could hardly be trusted with opportunities of practising the elaborate and extensive operations so thoroughly expounded in the former. For the first half of the volume is unsatisfactory in several ways. Classification and subdivision are pushed to an excess, presumably for the benefit of the student; thus seven varieties of oophoritis are described, exclusive of tuberculosis. Practical utility is, indeed, throughout subordinated to examination shibboleth to an extent which renders all these sections useless to the practitioner who is called upon to diagnose and treat a gynæcological case. Thus pelvic peritonitis and cellulitis are dismissed in five pages, compared with 52 allotted to displacements of the uterus; deciduoma malignum is discussed at greater length than is carcinoma of the cervix; about dysmenorrhœa are set down a few remarks on ætiology, but none whatever on symptoms and treatment; no description is given at all of abscess of Bartholin's gland, and of cysts in that organ we read less than of the curiosity known as kolpo-hyperplastica cystica of Winckel. When the author has brought the standard of the first half of this book up to that of the second and to that of his "Manual of Midwifery," we shall feel more justified in recommending it.

A Text-book of Treatment (alphabetically arranged). Part I., Obstetrics and Gynæcology. By John Campbell, M.A., M.D., F.R.C.S., Eng., Surgeon Samaritan Hospital for Women, Belfast. Pp. 279. Illustrations 93. (London: Sidney Appleton. Price 10s. net.)

This book is one of three parts of a text-book of treatment, the others being termed "Medicine" and "Surgery." It is strictly a Dictionary in that it is alphabetically arranged; in this method of presenting a subject either much repetition is necessary and occurs, or else reference has to be made to other portions of the book under some headings. For instance, under the heading "Endometritis" the reader is referred to "Metritis," and various methods are described, without indication as to which are more particularly applicable to endometritis or which to metritis. Apart from the disadvantages of the manner of dealing with his material, the author presents his teaching in a clear and readable manner, though his views are not all shared by gynæcologists in London. For instance, the wire snare is recommended for the removal of fibroid polypi, a method which is now practically discarded in favour of simple division of the pedicle with scissors. In cases where fibroids have been nucleated from the broad ligament, if the cavity is large drainage through the abdominal wall is advised. We should have thought it much better surgery to remove the cervix and allow the cavity to drain into the vagina. In the description of abdominal hysterectomy for cancer we think Wertheim's description might have been more closely followed with advantage, for the author does not make it at all clear how the ureters are to be attacked: especially so as the author remarks that the operation is practically that of Wertheim. In treating post-partum hæmorrhage it is advised to remove the placenta at once; if hæmorrhage then continues the author suggests looking for lacerations in the cervix, and if these are not found, methodically scraping the uterine cavity with a flushing curette and hot saline solution. We cannot but feel that such teaching as this is wrong, when it is realised how rare hæmorrhage from laceration is, and how easy it is as a rule to induce good retraction and contraction by bi-manual compression, which the author does not mention at all. Again, we do not like his management of the third stage of labour; he recommends waiting half an hour, and if the placenta is not then born gives instruction how to remove it. He omits to mention the signs, so well known, that mark the moment when the placenta leaves the uterus and slips into the vagina, the real moment when it is proper to remove the placenta without any regard to time. We regret to see that caustics, probes, Braun's syringe, etc., are still recommended for the treatment of cervical erosions and endometritis, with or without preliminary curettage. If curettage is done thoroughly and aseptically these methods are unnecessary; if curettage is not done, in our opinion they are futile. The illustrations are clear and very well done, and we are glad to see that rubber gloves are conspicuous in them.

### OPHTHALMOLOGY.

Ophthalmia Neonatorum, with Especial Reference to its Causation and Prevention. By Sydney Stephenson, M.B., C.M., Ophthalmic Surgeon to Queen Charlotte's Hospital, London. Pp. 258. (London: George Pulman and Sons.)

A comprehensive monograph on the subject of ophthalmia neonatorum, such as the work under review, is of great value, for though the disease is much less prevalent than formerly, mainly owing to the very widespread adoption of Credé's method of prophylaxis, the time has certainly arrived when the situation may be again surveyed and statistics examined. All figures go to prove that the percentage incidence of ophthalmia neonatorum has much diminished since the institution of a regular method of prophylaxis. This is a cause for congratulation, as it appears from the Report of the Royal Commission on the Blind, Deaf and the Dumb, in 1889, that about 7,000 persons in the United Kingdom were blind from this disease.

The ætiology of ophthalmia neonatorum is very fully discussed by the author, and it appears that the gonococcus of Neisser is responsible for approximately 67 per cent. of all cases of ophthalmia in the new-born, and that the ordinary pyogenic bacteria are responsible for nearly all the rest. Rarer organisms have been met with, but the cases are of hardly more than academic interest. The method of prophylaxis known as Credé's is most carefully surveyed in a very fair spirit, and an interesting table on p. 206 brings out the fact that the percentage of ophthalmia in cases treated with 1 per cent. silver nitrate is much lower than that occurring in cases treated with the 2 per cent. solution originally advocated by Credé. The author himself recommends the former, or as an alternative a 1 : 4000 solution of perchloride of mercury or other similar antiseptic. It is freely acknowledged that any of these agents may cause an inflammatory reaction in the majority of cases, and it seems probable that infection of the eyes during this period and not immediately at birth is responsible for the cases of ophthalmia which do occur in most maternity charities where the patients are visited in their own homes, and where the care of the nurse is powerless to prevent occasional infection owing to the parents' neglect or ignorance.

In treatment nothing new is suggested; Mr. Stephenson personally prefers protargol 10-50 per cent. or argyrol 25 per cent., though it is well known that some competent observers consider both these preparations as "inert fluids." Probably painting the lids with 1 or 2 per cent. silver nitrate solution and frequent antiseptic irrigation is still the most reliable method, though in gonorrhœal cases vaccine treatment deserves a thorough trial.

Admirable as is this work, one word of complaint may be made in conclusion. Is it necessary to incorporate all references in the body of the text, instead of relegating them to footnotes? In this small volume there are nearly 3,000 references to names of authors or journals, all in the text, the number rising in some places to thirty or more on a page. If such references were made as footnotes the value of the work would be undiminished and the flow of the text would be unimpeded.

Tests and Studies of the Ocular Muscles. By Ernest E. Maddox, M.D., F.R.C.S., Ed. Second Edition. (Philadelphia, U.S.A.: The Keystone Publishing Company. 255 pages, with 110 illustrations. Price 6s. 3d.)

The first edition of this book has been revised and enlarged. There is a new chapter on Nystagmus, which contains little that is new on the subject. Under the heading "Treatment of Nystagmus" we are told to "operate as early in life as possible for . . . corneal leucomato" (*sic*). The author does not suggest what operation to perform. We do not agree with the statement that "tenotomy or advancement might do good in exceptional cases." In the chapter on strabismus the author rightly emphasises the importance of the early treatment of squint. Any trouble taken in this way is well repaid. Much of the subject-matter in the book is very scientific, and will have little interest for the purely clinical reader. Some of the illustrations could be considerably improved. The book, however, is well worth a careful study.

### PHARMACOLOGY AND THERAPEUTICS.

The Chemical Basis of Pharmacology : An Introduction to Pharmacodynamics Based on the Study of the Carbon Compounds. By Francis Francis, D.Sc., Ph.D., Professor of Chemistry, Victoria College, Bristol, and J. M. Fortescue-Brickdale, M.A., M.D., Physician, Bristol Hospital for Sick Children, and Demonstrator of Physiology, University College, Bristol. (London : Edward Arnold. 8vo. Pp. vii+372. Price 14s. net.)

This is a work of considerable complexity, distinctly able and erudite, and scientifically of great use, but probably too difficult for most of us who have already forgotten a good deal of our organic chemistry.

The authors deal solely with organic compounds, giving full diagrams of the constitutional formulæ of each, and tracing the changes in therapeutical effects that result from the introduction of different radicles into the same fundamental carbon chain or ring. It is, of course, impossible to predict with absolute certainty what physiological effect an organic compound of given composition has; but there are certain broad rules that have been found to hold good in most cases. In a general sense, for example, Dujardin-Beaumetz and Bardel's conceptions of the influence of various side groups on the benzene compounds may be taken as accurate—namely, that—

1. Those containing hydroxyl are antiseptic.
2. Those containing an amido group or an acid amide are hypnotic.
3. Those containing both an amine group and an alkyl group are analgesic.

These few general rules, and others like them, have been elaborated in the book before us, and the various known compounds are discussed in respect of the extent to which their known physiological action, as tested experimentally, agrees with the action that theory would lead one to expect them to have. The authors have, we think, tackled a very difficult theme in a very able manner, and the book they have produced is almost the only one in which the subject has been dealt with in English since Sir Lauder Brunton's Croonian Lectures in 1889.

The perusal of the work shows the lines upon which the large manufacturers, aided by skilled chemists, have been able to invent such drugs as aspirin and mesotan, phenazone and phenacetin, and so forth. There is no doubt that science is here doing a great deal to assist empiricism; and although the market at the same time becomes flooded with scores of other drugs that are useless, it is partly the fault of the medical profession that the useless are not at once sorted from the useful; for the probable physiological action of an organic chemical drug may often be fairly accurately estimated by a consideration of its chemical structure. We lay the book down with a feeling of pity for the medical student of the future; for we are impressed with the idea that successful modern therapeutics will require a knowledge of many of the difficult things that are included in the volume.

### PATHOLOGY.

The Essentials of Cytology. By C. E. Walker, Assistant Director of the Cancer Research, Liverpool; Honorary Lecturer in Cytology to the School of Tropical Medicine, Liverpool. 8vo. Pp. 139, with 49 illustrations. (London : Archibald Constable and Co., Limited. Price 7s. 6d. net.)

Cytology, though a branch of biological science that does not concern the medical man in his daily work, is yet one which concerns him very closely. The practitioner, it is true, prefers that others should carry out the research work and that he should receive a report from time to time upon its practical issues. Nevertheless, it is always interesting to see the lines upon which biological work is being carried on; and the volume before us, though it contains nothing that can be turned to immediate and direct account in connection with the patients one may be seeing daily, is a most interesting summary of our present knowledge of the anatomy and physiology of the individual cells of which all living matter is built up. The author points out that there was once a time when scientists believed that the physical basis of life was protoplasm. It is now known that no homogeneous mass of protoplasm is really alive. The phenomena exhibited by living matter can only be exhibited by protoplasm which is in the form of cells or a cell. The cell, as a whole, with all its constituent parts, is essential to life; and all living things, whether animal or vegetable, are only alive when their protoplasm is highly differentiated in the cells of which the living structure is built up. The cell as a whole, and not merely the protoplasm in it, is the unit of living matter. The problems of living matter—whether those problems be physiological (normal) or pathological (abnormal)—ultimately resolve themselves into cell problems. The importance of knowing everything that can be discovered about the structure of the cell—its wall, its protoplasm, its nucleus, its nucleolus, its centrosome, its every part, and about the functions of the cell; its modes of division, its metabolism, and so forth, and its chemical composition—at once becomes very obvious. The profession must be all the more grateful to those who are devoting their time and their brains to learning more about the ultimate cell, and who, having worked thus, take the trouble to tell what they know in as clear a way as has been done in this book. Methods of cytological research are given at some length. The relations between Mendel's law of heredity and the behaviour of chromosomes of dividing cells are described. The diagrams are easy to follow. The paper is good and the type excellent. There is a curious addendum in a pocket attached to the last cover, namely, a series of eight stereoscopic views illustrating the three-dimensional nature of the process of mitotic cell-division.

Medical Laboratory Methods. By Herbert French, M.D., F.R.C.P., Assistant Physician to Guy's Hospital, etc. Second edition. 1908. Pp. viii. + 168. Price 5s. net. (London : Baillière, Tindall, and Cox.)

A more striking example of the value to a book of various accessories which might be thought immaterial could hardly be selected than this second edition of Dr. French's clinical laboratory handbook. Improved paper, the judicious use of distinctive type, and, above all, the substitution of good for inferior illustrations have now been combined with the clear and practical letterpress of a work which has filled a distinct gap, and will fill it more worthily still in the future. Throughout the book revision of old matter and the inclusion of new is evident in all directions, and a special chapter has been inserted on the preservation and staining of morbid anatomy specimens. Coloured plates of various micro-organisms and of the varieties of normal and abnormal blood corpuscles are also a considerable gain. If one were in a cavilling mood it might be suggested that the former of these would be better placed in the chapter dealing with the examination of pus rather than amidst the chemical examination of the gastric contents, where it now stands. But, after all, cavilling is quite unnecessary in the case of so excellent a manual as this, whose author has succeeded in keeping within the limits he has marked out for himself with a result that is wholly admirable.

